# Supplementary material for: HCV genotype profile in Brazil of mono-infected and HIV co-infected individuals: A survey representative of an entire country
Source: PLoS One. 2020 Jan 6;15(1):e0227082. doi: 10.1371/journal.pone.0227082 (PMC6944355; doi:10.1371/journal.pone.0227082)
Supplement: Attachment — Submitted filename: PONE-D-19-24177_reviewer (1).pdf [file pone.0227082.s001.pdf]

PLOS ONE

HCV genotype profile in Brazil of mono-infected and HIV co-infected individuals: a survey representative of an entire country

--Manuscript Draft--

**Manuscript Number:**	PONE-D-19-24177
**Article Type:**	Research Article
**Full Title:**	HCV genotype profile in Brazil of mono-infected and HIV co-infected individuals: a survey representative of an entire country
**Short Title:**	HCV genotype profile in Brazil of mono-infected and HIV co-infected individuals
**Corresponding Author:**	Ricardo Sobhie Diaz<br>Federal University of São Paulo<br>Sao Paulo, SP BRAZIL
**Keywords:**	Hepatitis C genotype; HCV/HIV co-infection; Brazil
**Abstract:**	Abstract Introduction: To be eligible for government-provided treatment in Brazil, all HCV-infected patients are required to be genotyped shortly after diagnosis. We assessed HCV genotype (G) profiles according to geographic region, gender, age and HIV co-infection. Methods: We assessed 29,071 genotypes collected from HCV-infected patients from March 2016 to March 2018 (Abbott Real-Time HCV Genotype). We tested 12,336 randomly selected samples for HIV co-infection using an EIA rapid test kit (TR DPP HIV 1/2 Bio-Manguinhos). Descriptive statistical analyses were performed using R. Results: Overall, HCV G distribution was 40.9% G1A, 30.2% G1B, 23.8% G3, 3.8% G2, 0.7% G4, 0.1% G5 and 0.6% with multiples genotypes. G1A prevalence was 44.4% among males and 35.8% among females. G1B and G2 were more prevalent in older individuals than G1A and G3. G3 was more prevalent in the South region. Of samples tested for HIV co-infection, 15% were HIV+. Median age of HIV co-infected was 50 years compared to 57 years among mono-infected individuals (71.1% males compared to 58.7%). Distinct prevalence between co-infected and mono-infected individuals were respectively: G1A 60.6% versus 37.8%, G1B 15.2% versus 32.9%, and G3 18.9% versus 24.7%. G4 was detected among co-infected young men (3.5% versus 0.2% among mono-infected). Conclusion: The increasing prevalence of G3, as inferred by young ages, poses extra challenge related to disease progression. Distinct G profiles between mono and HIV co-infected warrants future research in order to understand and mitigate HCV chain of transmission.
**Order of Authors:**	Mariana Fernanda Rodrigues Nutini
	James Hunter
	Leila Giron
	Ana Flavia Nacif Pinto Coelho Pires
	Igor Massaki Kohiyama
	Michelle Camargo
	Maria Cecilia Araripe Sucupira
	Adele Schwartz Benzaken
	Paulo Abrão Ferreira
	Hong-Ha M. Truong
	Ricardo Sobhie Diaz
**Additional Information:**	
**Question**	**Response**
**Financial Disclosure**	HCV genotype is provided by the Brazilian Ministry of Health. HIV rapid tests were donated to the authors by the Brazilian Ministry of Health. MN was supported by grants

Powered by Editorial Manager® and ProduXion Manager® from Aries Systems Corporation

Enter a financial disclosure statement that describes the sources of funding for the work included in this submission. Review the submission guidelines for detailed requirements. View published research articles from *PLOS ONE* for specific examples.<br><br>This statement is required for submission and **will appear in the published article** if the submission is accepted. Please make sure it is accurate.<br><br>**Unfunded studies**<br>Enter: *The author(s) received no specific funding for this work.*<br><br>**Funded studies**<br>Enter a statement with the following details:<br>• Initials of the authors who received each award<br>• Grant numbers awarded to each author<br>• The full name of each funder<br>• URL of each funder website<br>• Did the sponsors or funders play any role in the study design, data collection and analysis, decision to publish, or preparation of the manuscript?<br>• **NO** - Include this sentence at the end of your statement: *The funders had no role in study design, data collection and analysis, decision to publish, or preparation of the manuscript.*<br>• **YES** - Specify the role(s) played.<br><br>* typeset	from CAPES (Coordenação de Aperfeiçoamento de Pessoal de Nível Superior), Brazil.
**Competing Interests**<br><br>Use the instructions below to enter a competing interest statement for this submission. On behalf of all authors, disclose any competing interests that could be perceived to bias this work—acknowledging all financial support and any other relevant financial or non-financial competing interests.<br><br>This statement **will appear in the published article** if the submission is accepted. Please make sure it is accurate. View published research articles from *PLOS ONE* for specific examples.	The authors have declared that no competing interests exist.

Powered by Editorial Manager® and ProduXion Manager® from Aries Systems Corporation

**NO authors have competing interests**<br><br>Enter: *The authors have declared that no competing interests exist.*<br><br>**Authors with competing interests**<br><br>Enter competing interest details beginning with this statement:<br><br>*I have read the journal's policy and the authors of this manuscript have the following competing interests: [insert competing interests here]*<br><br>* typeset	
**Ethics Statement**<br><br>Enter an ethics statement for this submission. This statement is required if the study involved:<br><br>• Human participants<br>• Human specimens or tissue<br>• Vertebrate animals or cephalopods<br>• Vertebrate embryos or tissues<br>• Field research<br><br>Write "N/A" if the submission does not require an ethics statement.<br><br>General guidance is provided below. Consult the submission guidelines for detailed instructions. **Make sure that all information entered here is included in the Methods section of the manuscript.**	This study was approved by the Ethical Committee of the Federal University of São Paulo (#1687/2016).

**Format for specific study types**<br><br>**Human Subject Research (involving human participants and/or tissue)**<br>• Give the name of the institutional review board or ethics committee that approved the study<br>• Include the approval number and/or a statement indicating approval of this research<br>• Indicate the form of consent obtained (written/oral) or the reason that consent was not obtained (e.g. the data were analyzed anonymously)<br><br>**Animal Research (involving vertebrate animals, embryos or tissues)**<br>• Provide the name of the Institutional Animal Care and Use Committee (IACUC) or other relevant ethics board that reviewed the study protocol, and indicate whether they approved this research or granted a formal waiver of ethical approval<br>• Include an approval number if one was obtained<br>• If the study involved *non-human primates*, add *additional details* about animal welfare and steps taken to ameliorate suffering<br>• If anesthesia, euthanasia, or any kind of animal sacrifice is part of the study, include briefly which substances and/or methods were applied<br><br>**Field Research**<br><br>Include the following details if this study involves the collection of plant, animal, or other materials from a natural setting:<br>• Field permit number<br>• Name of the institution or relevant body that granted permission	
**Data Availability**<br><br>Authors are required to make all data underlying the findings described fully available, without restriction, and from the time of publication. PLOS allows rare exceptions to address legal and ethical concerns. See the PLOS Data Policy and FAQ for detailed information.	Yes - all data are fully available without restriction

A Data Availability Statement describing where the data can be found is required at submission. Your answers to this question constitute the Data Availability Statement and **will be published in the article**, if accepted.<br><br>**Important:** Stating 'data available on request from the author' is not sufficient. If your data are only available upon request, select 'No' for the first question and explain your exceptional situation in the text box.<br><br>Do the authors confirm that all data underlying the findings described in their manuscript are fully available without restriction?	
**Describe where the data may be found in full sentences. If you are copying our sample text, replace any instances of XXX with the appropriate details.**<br><br>• If the data are **held or will be held in a public repository**, include URLs, accession numbers or DOIs. If this information will only be available after acceptance, indicate this by ticking the box below. For example: *All XXX files are available from the XXX database (accession number(s) XXX, XXX.).*<br>• If the data are all contained **within the manuscript and/or Supporting Information files**, enter the following: *All relevant data are within the manuscript and its Supporting Information files.*<br>• If neither of these applies but you are able to provide **details of access elsewhere**, with or without limitations, please do so. For example:<br><br>*Data cannot be shared publicly because of [XXX]. Data are available from the XXX Institutional Data Access / Ethics Committee (contact via XXX) for researchers who meet the criteria for access to confidential data.*<br><br>*The data underlying the results presented in the study are available from (include the name of the third party*	Data are held or will be held in a public repository, and information will be provided upon acceptance of manuscript.

and contact information or URL).<br>• This text is appropriate if the data are owned by a third party and authors do not have permission to share the data.<br><br>* typeset	
Additional data availability information:	Tick here if the URLs/accession numbers/DOIs will be available only after acceptance of the manuscript for publication so that we can ensure their inclusion before publication.

Manuscript

Click here to download Manuscript Diaz, RSD. HCV HIV MN 18.docx

HCV genotype profile in Brazil of mono-infected and HIV co-infected individuals: a survey representative of an entire country

Mariana Fernanda Rodrigues Nutini[1], James Hunter[1], Leila Giron[1], Ana Flavia Nacif Pinto Coelho Pires[2], Igor Massaki Kohiyama[2], Michelle Camargo[2], Maria Cecilia Araripe Sucupira[1], Adele Schwartz Benzaken[2], Paulo Abrão Ferreira[1], Hong-Ha M. Truong[3], Ricardo Sobhie Diaz[1]

[1]Federal University of Sao Paulo, Brazil.

[2]Sexually Transmitted Infections/AIDS and Viral Hepatitis Department of the Brazilian Ministry of Health.

[3]Department of Medicine, University of California, San Francisco, USA

Keywords: Hepatitis C genotype; HCV/HIV co-infection; Brazil

Running Title: HCV genotype in Brazil

Corresponding Author: rsdiaz@catg.com.br

Article's main point: HCV genotype from many newly diagnosed Brazilians were determined. Results suggested increasing prevalence of genotypes 1A (HCV mono and HCV/HIV co-infected) and genotype 3 (HCV mono-infected) and revealed unprecedent emergency of Genotype 4 among young HIV-infected males.

Abstract

Introduction: To be eligible for government-provided treatment in Brazil, all HCV-infected patients are required to be genotyped shortly after diagnosis. We assessed HCV genotype (G) profiles according to geographic region, gender, age and HIV co-infection.

Methods: We assessed 29,071 genotypes collected from HCV-infected patients from March 2016 to March 2018 (Abbott Real-Time HCV Genotype). We tested 12,336 randomly selected samples for HIV co-infection using an EIA rapid test kit (TR DPP HIV 1/2 Bio-Manguinhos). Descriptive statistical analyses were performed using R.

Results: Overall, HCV G distribution was 40.9% G1A, 30.2% G1B, 23.8% G3, 3.8% G2, 0.7% G4, 0.1% G5 and 0.6% with multiples genotypes. G1A prevalence was 44.4% among males and 35.8% among females. G1B and G2 were more prevalent in older individuals than G1A and G3. G3 was more prevalent in the South region. Of samples tested for HIV co-infection, 15% were HIV+. Median age of HIV co-infected was 50 years compared to 57 years among mono-infected individuals (71.1% males compared to 58.7%). Distinct prevalence between co-infected and mono-infected individuals were respectively: G1A 60.6% versus 37.8%, G1B 15.2% versus 32.9%, and G3 18.9% versus 24.7%. G4 was detected among co-infected young men (3.5% versus 0.2% among mono-infected).

Conclusion: The increasing prevalence of G3, as inferred by young ages, poses extra challenge related to disease progression. Distinct G profiles between mono and HIV co-infected warrants future research in order to understand and mitigate HCV chain of transmission.

Introduction

Brazil is a large "continental size" country divided into five macro-regions: North, Northeast, Central, Southeast, and South. Prevalence of HCV infection varies according to different regions among adults ≥18 years old, ranging from 3.2% in the North, to 1.9 in the Central and South, 1.8 in the Southeast, and 1.6 in Northeast. [1] HCV diagnostic and monitoring testing as well as treatment is fully paid for by the Brazilian Public Health system, as all citizens ≥18 years old are entitled to receive treatment according to local guidelines. The Brazilian national protocol for treatment uses the combination sofosbuvir/ledipasvir (patients without previous treatment with G1), sofosbuvir/velpatasvir (patients without previous treatment with non-G1) and glecaprevir/pibrentasvir + sofosbuvir (retreatment). (http://conitec.gov.br/images/Relatorios/2018/Relatorio_PCDT_HepatiteC.pdf).

HCV/HIV co-infection is of particular concern since liver fibrosis progression rate in this population is more rapid. [2, 3] HIV viremia is an independent predictor of liver fibrosis progression among these pati[illegible]

Contemporary charact[illegible]ies on the genetic profile of these ag[illegible]by phylogenetic analysis. Therefor[illegible] genotypes or types, with genetic similarities ranging between 66 to 69%, sub-genotypes or subtypes with similarities ranging from 77 to 80%, and the so-called viral quasispecies with similarities ranging from 91 to 99%. [5] The emerging viral pathogen is thought to evolve genetically in the host forming the viruses quasispecies. By founder effect, new diverse viral pathogens may determine the genetic profile of the virus in a given population or geographic region. It is also

> A general description of genetic diversity instead of HCV diversity?

conceivable that distinct viral types or subtypes, as distinct biologic entities, may cause distinct pathogenicity, transmissibility or treatment response to antivirals. As a classical example, disease progression, response to antiretroviral treatment and transmissibility are distinct between HIV-1 and HIV-2. [6]

It has been demonstrated that HCV is divided into ~~seven~~ eight genotypes with ~~several~~ 90 sub-genotyp[illegible]c, 2, 3, 4, 5, and 6. [7] En[illegible] include genotypes 1 and 4 in Central Africa, 2 in West Africa, 5 [illegible] Central/Southern Africa, and 3 and 6 in the South and East As[illegible] subtypes of endemic regions have spread and become establis[illegible] of the globe by founder effect. Th[illegible] the developed world have spread widely a[illegible]ons of blood components, [illegible]g use. [13-15]

in America

developed world…and in the others countries?

The genotype ? or the mutations. What about the pangenotypic treatment?

HCV genotype is of importance, since th[illegible] direct antiviral agents (DAAs) is usually lo[illegible] As an example, the natural polymorphisms [illegible] also be related to decrease response to sime[illegible]. [17, 18] Genotype 3 may harbor natural polymorphisms that may impair response to NS5A antagonists in the presence of cirrhosis. [19] Although pangenotypic DAAs have been developed, such as the combination of sofosbuvir and daclatasvir, sofosbuvir and velpatasvir, glecaprevir and pibrentasvir, treatment still need to be adjusted among cirrhotic patients as we see for genotype 3, as well as the duration of treatment using sofosbuvir and daclatasvir or use of ribavirin for sofosbuvir and velpatasvir. Besides guiding treatment decisions, the evaluation of the molecular

However, this manuscript is about genotypes not polymorphisms

biology dynamics in a given region can provide clues about the expansion of a given strain, conceivably helping inform the design of future vaccines.

As of January 2016, all HCV genotype determination for the Brazilian Minister of Health Program is performed at a centralized molecular biology laboratory (Laboratorio Centro de Genomas, São Paulo, Brazil). Individuals with recent HCV diagnoses by serology will have a HCV viral load as a confirmatory test and a genotype test, followed by a liver fibrosis evaluation. We present the first reported results of HCV genotype profile among individuals recently diagnosed with HCV according to Brazilian geographic region, gender, age and HIV co-infection.

Methods

Samples from 29,071 HCV recently diagnosed individuals from entire Brazil country collected from March 2016 to March 2018 were analyzed. To assess the prevalence of HCV/HIV co-infection, 12.336 samples were randomly selected for HIV testing. As recommended by the World Health Organization for surveillance studies for HIV prevalence, one EIA test is sufficient if the HIV prevalence in the given population is greater than 10%. [20] This study was approved by the Ethical Committee of the Federal University of São Paulo (#1687/2016).

The samples received were tested for HCV genotype using the commercial Abbott kit according to the manufacturer's specifications (https://www.abbottmolecular.com/products/realtime-hcv-genotype-II.html). Briefly, the *Abbott Real Time HCV genotype II* is an in vitro RT-PCR assay using the polymerase chain reaction for the determination of HCV genotypes in the plasma or serum of individuals infected with this virus. The assay detects genotypes 1, 2, 3, 4, 5

and 6, and subtypes 1a and 1b through the use of fluorescent oligonucleotide probes with specific labeling for each genotype. For samples which genotype assignment was unclear using qPCR, Sanger sequencing of either NS5B or 3´UTR regions were performed. [21]

All samples submitted to HCV subtyping by MS were tested for HIV antibodies using the rapid *HIV test kit TR DPP® HIV1/2, Bio-Manguinhos - Institute of Technology in Immunobiology, Industry,* in accordance with the manufacturer's instructions. The test qualitatively determines the presence of the anti-HIV antibody by immunochromatographic method, is easy to perform, does not require laboratory infrastructure for its performance and can generate results within 30 minutes. [22]

The data was organized and analyzed using the R Statistical Computing System on a Macintosh (version 3.5.1). [23] Initial data cleaning and graphics used the Tidyverse set of packages for R. [24] Descriptive statistics were performed with the DescTools package and group comparisons conducted by chi-squared analytic algorithms in the base R system and the gmodels package. [25, 26]

Results

There was a large representation of women in this survey, as 41.3% of the 29,071 individuals genotyped were female. There was also a reasonable representation of each Brazilian Macro-region, which in general reconciles with general population number among these regions. We evaluated 8,012 individuals from the South region (27,6%), 15.767 from the Southeast region (54,2%), 1.128 from Center (3,9%), 2.796 from the northeast (9,6%) and 1.368 from the North region (4,7%). As seen in Table 1, the current overall prevalence of genotype profile was

highest for G1A at 40.9%, followed by G1B at 30.2%, and G3 at 23.8%, with the prevalence of other genotypes at less than 4%.

Overall							
	Genotypes						
	G1 A	G1 B	G2	G3	G4	G5	Multiple
(%)	40.9	30.2	3.8	23.8	0.7	0.1	0.6
median Age (55)	52	60	61	56	45	63	56
Male	44.4	26.1	3.5	24.2	0.9	0.1	0.7
median Age Male (54)	52	59	60	55	44	71	56
Female	35.8	36.0	4.1	23.1	0.3	0.2	0.6
median Age Female (57)	53	60	61	57	54	56	54
HCV Mono-infected							
(%)	37.8	32.9	3.7	24.7	0.2	0.1	0.6
median Age (57)	54	61	63	57	56	63	58
Male	40.9	29.1	3.6	25.3	0.2	0.1	0.8
median Age Male (56)	53	60	62	56	50	71	58
Female	33.5	38.0	3.9	23.9	0.2	0.2	0.5
median Age Female (58)	55	62	63	58	63	56	63
HIV co-infected							
15,0% prevalence							
(%)	60.6	15.2	2.2	18.9	2.5	0.0	0.6
median Age (50)	49	51	54	51	38		50
Male	61.7	13.7	2.3	18.1	3.5	0.0	0.7
median Age Male (49)	49	51	54	50	38		52
Female	57.6	19.1	1.9	20.9	0.0	0.0	0.4
median Age Female (50)	50	52	54.5	52			45

Table 1. Demographic data of HCV mono and HIV co-infected individuals describing HCV genotype according to gender and age.

We explored whether median age correlates with the expansion dynamics of the epidemic: the lower the age, the greater the expansion of a particular genotype or specific status (gender, co-infection, etc.). The overall mean age for males was 54.1 years old, which was significantly lower than 56.1 years old for females (Welch's Two Sample t-test, $t = 13.367$, $df = 23251$, $p < 10^{-6}$). The median age for males was 54 and for females was 57. Overall, the mean age was significantly lower among

individuals infected by G1A, G3 and G4 f[illegible]$^{-6}$) as shown in Table 1. When considering just G4, the m[illegible] was significantly lower than 54 for fem[illegible]750, df = 38.5, p = 0.0045).

This analysis could be also in relation of risk factors of patients

As shown in Table 3, G1A was more prevalent in the South, Southeast and Central regions, with G1B being more prevalent in the Northern regions ($p < 10^{-6}$). Of note, G3 is more prevalent than G1B in the South region (Chi-squared test, χ^2 = 4059.6, df = 1, $p < 10^{-6}$), as this genotype has a similar prevalence as G1 among females in this region (Table 3).

Subtype	Males		Females	
	Mean	Median	Mean	Median
G1A, G3 & G4	52.47	53	53.97	55
G1B, G2, G5 & Multiples Genotypes	57.91	59	59.12	61
t-test value	-28.284		-21.432	

Table 3: Difference in Median Ages by Gender for Groupings of Subtypes

Prevalence of HIV co-infection among patients with HCV was 15.0%. HCV/HIV co-infection prevalence was higher among men compared to women (18.1% versus 10.4%, Chi-squared test, χ^2 = 140.3, df = 1, $p < 10^{-6}$), and the mean

age for HIV co-infected individuals was lower compared to persons mono-infected (Welch's Two Sample t-test, t = 28.1, df = 3069, $p < 10^{-6}$ (Table 2).

After detection of HIV seropositivity, we cross-checked the Brazilian National Database to confirm if the infected patients had been reported to the system. The reporting of HIV infection is a requirement of the Brazilian Ministry of Health in order to provide monitoring testing and antiretroviral treatment to the HIV infected individuals. According to the National data base, 9% of individuals have not been previously diagnosed with HIV infection.

HIV/HCV co-infection prevalence varied by geographic regions, ranging from 19.1% in the South, to 15.6% in the Southea[illegible]ortheast, 10.9% in the Central region, and 4.3% in the [illegible] $< 10^{-6}$). As seen in Table 2, co-infection wa[illegible]nfected by G1A: 60.6% among co-infected and 37.8% among mono infected (One Sample Proportions test, $\chi^2 = 1584.4$, df = 1, $p < 10^{-6}$). It was also lower for G1B: 15.2% among co-infected compared to 32.9% of mono infected ($\chi^2 = 1584.4$, df = 1, $p < 10^{-6}$). G3 was less prevalent among HIV co-infected individuals (18.9%) than among mono-infected patients (24.[illegible] prevalence was still[illegible]

any association of risk factors?

No differences of G4 prevalence in monoinfected and coinfected patients?

**Overall**							
	**Genotypes**						
	**G1 A**	**G1 B**	**G2**	**G3**	**G4**	**G5**	**Multiple**
**Total**	**40.9**	**30.2**	**3.8**	**23.8**	**0.7**	**0.1**	**0.6**
**(%) males/females**	**44,4/35.8**	**26.1/36,0**	**3.5/4.1**	**24.2/23.1**	**0.9/0.3**	**0.1/0.2**	**0.7/0.6**
**Regions**							
**North**	**26.2**	**44.7**	**5.4**	**22.8**	**0.1**	**0.1**	**0.8**
**Northeast**	**31.6**	**42.3**	**2,8**	**22.4**	**0.4**	**0**	**0.5**
**Central**	**50.7**	**25.7**	**2.3**	**19.9**	**0.7**	**0**	**0.7**
**Southeast**	**42.1**	**33.5**	**3**	**19,7**	**1**	**0.1**	**0.5**
**South**	**42.7**	**17.7**	**5.5**	**33**	**0.2**	**0**	**0.9**
**HCV Mono-infected**	**37.8**	**32.9**	**3.72**	**24.7**	**0.19**	**0.09**	**0.6**
**(%) males/females**	**40.9/33.5**	**29.1/38.0**	**3.6/3.9**	**25.3/23.9**	**0.2/0.2**	**0.1/0.2**	**0.8/0.5**
**Regions**							
**North**	**24.6**	**45.6**	**6.3**	**22.8**	[illegible]	**0.2**	**0.5**
**Northeast**	**30.4**	**43.1**	**2.7**	[illegible]	[illegible]	[illegible]	**0.6**
**Central**	**49.1**	**25.4**	**2.2**	[illegible]	[illegible]	[illegible]	**0.2**
**Southeast**	**38.7**	**36.5**	[illegible]	[illegible]	[illegible]	[illegible]	**0.7**
**South**	**39.8**	**19.1**	[illegible]	[illegible]	[illegible]	[illegible]	**0.8**
**HIV co-infected**	**60.6**	**15.2**	[illegible]	[illegible]	[illegible]	[illegible]	[illegible]
**(%) males/females**	**61.7/57.6**	**13.7/19.1**	[illegible]	[illegible]	[illegible]	[illegible]	[illegible]
**Regions**							
**North**	**48**	**20**	[illegible]	[illegible]	[illegible]	[illegible]	[illegible]
**Northeast**	**46.4**	**34**	[illegible]	[illegible]	[illegible]	[illegible]	**1**
**Central**	**67.9**	**12.5**	[illegible]	[illegible]	[illegible]	[illegible]	**0**
**Southeast**	**61.3**	**17.2**	[illegible]	[illegible]	[illegible]	[illegible]	**0.5**
**South**	**61.4**	**9**	[illegible]	[illegible]	[illegible]	[illegible]	**0.6**

A map could be more illustrative of HCV genotype distribution

Table 2. HCV genotype prevalence according to Brazilian Geographic regions, gender, and HIV co-infection.

The overall median age in this survey was 55 years old, and was lower among HIV-infected individuals (50 years old) than HCV mono-infected individuals (57 years old). The comparison of means by a Welch's Two-Sample t-test produced a t-value of 28.147 on 3069 df with a p-value < 10^{-6}. The median age among men was lower compared to women in mono-infected individuals, but similar between genders in the co-infected individuals (Table 3). Also, we noted that the median age for individuals infected by G4 was significantly lower among HIV co-infected males (38 years old) compared to HCV mono-infected females (50 years old) (Kruskal-Wallis rank sum test, $\chi^2 = 7.7$, df = 1, p = 0.006). There were no HCV/HIV co-infected females with G4 in the study.

Discussion

The prevalence of distinct HCV genotypes in Brazil reveals an interesting dynamic suggesting establishment and expansion of strains considered in the recent past as minorities in the country. When analyzing the HCV prevalence according to regions, gender, age and HIV co-infection, one can speculate about the trends of the HCV genotype profile according probably to multiple founder effect phenomena.

For example, in contrast to what have been usually described in Brazil [27, 28], G1B is not the dominant genotype in Brazil overall. Prevalence of G1A was higher overall, with an increasing prevalence trend of G3 based on the lower ages of G3-infected individuals as compared to G1. Furthermore, the median age among individuals infected by G1A and G3 was lower than G1B and G2, suggesting that G1A and G3 are clearly expanding in the Br[illegible]here was a higher prevalence of G1A and [illegible] Central, South-east and South regi[illegible] northern regions of Brazil. Taking thes[illegible]CV epidemics tend to grow in the Southern regions of the country, and more attention should be given to these features in relation to attempts to interrupt transmission chains. Of note is the higher prevalence among women infected by G3 in the South region of Brazil.

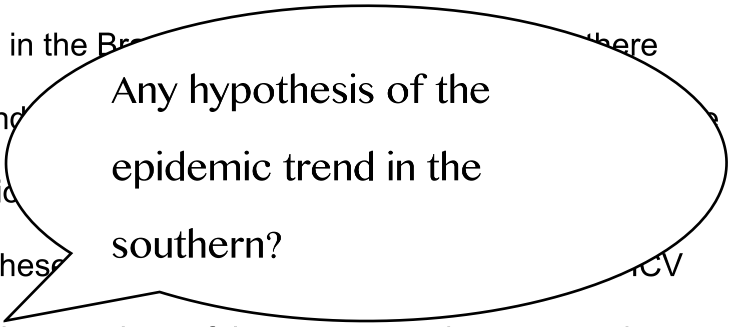

The current data also corroborates a very interesting study that used a phylogenetic model to infer the time for introducing specific HCV genotypes in Brazil [29]. According to the Time to Most Recent Common Ancestor (TMRCA) as inferred by Bayesian analysis, G1B entered in Brazil around the year 1923 (1844-1967), whereas G3 entered in the year 1967 (1955-1980), and G1A in 1979 (1967-1987). More interestingly, the inferred Growth rate (r) is 0.26 for G1B, 0.32 for G3 and 0.4

for G1A. These *r* values explain the finding why G1A became more prevalent than G1B and suggests that soon, G1B will also be overcome by G3. It is also conceivable that the once rare HCV G4 is, by founder effect, is growing extensively among HIV co-infected young men. This result points to the need to better understand this feature and relationships with mode of HCV acquisition such as among men that have sex with men, and attention should be given to interrupt this potential chain of transmission.

We also found a high prevalence of HIV co-infection among recently-diagnosed HCV-infected individuals (15%), as the co-infection prevalence was higher among men as compared to women. The HIV epidemic in Brazil is concentrated, however, the sex distribution of HIV infection have changed from 1 woman for 28 men in the mid-1980’s to 1 woman for about 1.5 men in 2017. The increased incidence among women emphasizes that women are becoming more vulnerable to HIV infection in Brazil. [30] Nonetheless, HCV co-infection is more prevalent among men in this current survey, which reconciles with international trends to high HCV incidence among HIV infected ~~man~~ men that have sex with men. [31, 32] We recognize that due to the nature of the current study, risk factors for HIV acquisition were not part of this survey, thereby precluding more definite conclusions.

We also observed that the median age among HIV co-infected individuals was lower than among HCV mono-infected individuals, which also suggest a growing epidemic among HIV-infected individuals. Therefore, efforts should target better HCV prevention strategies a[illegible]o of interest, prevalence of HIV co-infection was hi[illegible]pared to the North, suggesting a higher vulnerability to HCV infection in the South. Interestingly, Southern regions of Brazil are wealthier and more developed than the North.

vulnerability?
reasons?

The importance of the genetic diversity of a viral pathogen relies on the fact that genetically distinct strains may be considered as distinct biologic entities. For instance, longitudinal studies have demonstrated that G3 may significantly increase the HCV pathogenicity with respect to rates of steatosis, fibrosis, and hepatocellular carcinoma. It has been demonstrated that in comparison to other HCV genotypes, G3 may be associated to higher lipid levels and hepatic steatosis. [33] A higher rate of liver fibrosis over time is also associated to G3, with a 50% increased risk in G3-infected individuals compared to other genotypes. [34] It has also been described that individuals infected by G3 may have a 31% higher risk of developing cirrhosis and 80% higher risk of developing hepatocellular carcinoma in comparison to G1-infected individuals. [35] In this sense, the expansion of G3 HCV in certain areas of Brazil may constitute a very negative feature of this given epidemics.

Although the treatment response on interferon-based regimens is better among G3-infected individuals as compared to other genotypes, treatment with DAAs poses an additional challenge for individuals infected with G3. [36, 37] In general, G3 is not susceptible to first-generation HCV protease inhibitors such as telaprevir [38] Furthermore, when NS5A inhibitors are used, it has been demonstrated that polymorphisms previously present at the NS5A genomic region would impair the virologic response to treatment among cirrhotic patients. [39]

With regards to genotype 1, it has also been demonstrated that the genetic barrier to resistance to HCV protease inhibitors is lower for G1A compared to G1B, leading to a faster virologic failure among G1A-infected individuals. [40] Therefore, an increase in the prevalence of G1A and G3 poses an additional challenge for treatment with some DAAs.

We recognize that the prevalence of HIV co-infection may be overestimated if the HCV diagnostic is more frequently requested among individuals that have been already diagnosed as infected with HIV. Nonetheless, this is the largest report on HCV genotype in Brazil, which we believe it is truly representative of the entire country, showing an unprecedent growth of G1A and G3 in certain Brazilian regions and a very large prevalence of HIV co-infection among recently diagnosed individuals.

Funding: HCV genotype is provided by the Brazilian Ministry of Health. HIV rapid tests were donated to the authors by the Brazilian Ministry of Health. MN was supported by grants from CAPES (Coordenação de Aperfeiçoamento de Pessoal de Nível Superior), Brazil.

Conflict of Interest: Authors declare no conflict of interest

Acknowledgements: We thanks Fabiana Santos for helping with HIV rapid testing.

References:

1. Pereira LM, Martelli CM, Moreira RC, Merchan-Hamman E, Stein AT, Cardoso MR, et al. Prevalence and risk factors of Hepatitis C virus infection in Brazil, 2005 through 2009: a cross-sectional study. BMC Infect Dis. 2013;13(60). doi: 10.1186/1471-2334-13-60.
2. Benhamou Y, Bochet M, Di Martino V, Charlotte F, Azria F, Coutellier A, et al. Liver fibrosis progression in human immunodeficiency virus and hepatitis C virus coinfected patients. The Multivirc Group. Hepatology. 1999;30(4):1054-8.
3. Di Martino V, Rufat P, Boyer N, Renard P, Degos F, Martinot-Peignoux M, et al. The influence of human immunodeficiency virus coinfection on chronic hepatitis C in injection drug users: a long-term retrospective cohort study. Hepatology. 2001;34(6):1193-9.
4. Bräu N, Salvatore M, Ríos-Bedoya CF, Fernández-Carbia A, Paronetto F, Rodríguez-Orengo JF, et al. Slower fibrosis progression in HIV/HCV-coinfected patients with successful HIV suppression using antiretroviral therapy. J Hepatol. 2006;44(1):47-55. Epub 2005 Jul 27.
5. Fishman SL, Branch AD.The Quasispecies Nature and Biological Implications of the Hepatitis C Virus. Infect Genet Evol. 2009; 9(6): 1158–1167.
6. Marlink R, Kanki P, Thior I, Travers K, Eisen G, Siby T, et al. Reduced rate of disease development after HIV-2 infection as compared to HIV-1. Science. 1994; 265(5178):1587-90.
7. Nakano T, Lau GM, Lau GM, Sugiyama M, Mizokami M. An updated analysis of hepatitis C virus genotypes and subtypes based on the complete coding region. Liver Int, 2012, 32: 339–345.

8. Simmonds P. The origin of hepatitis C virus. Curr Top. Microbiol Immunol. 2013;369:1–15.
9. Jeannel D, Fretz C, Traore Y, et al. Evidence for high genetic diversity and long-term endemicity of hepatitis C virus genotypes 1 and 2 in West Africa. J Med Virol. 1998;55:92–7.
10. Candotti D, Temple J, Sarkodie F, Allain JP. Frequent recovery and broad genotype 2 diversity characterize hepatitis C virus infection in Ghana, West Africa. J Virol. 2003;77:7914–23.
11. Ndjomou J, Pybus OG, Matz B. Phylogenetic analysis of hepatitis C virus isolates indicates a unique pattern of endemic infection in Cameroon. J Gen Virol. 2003;84 Pt 9:2333–41.
12. Gower E, Estes C, Blach S, Razavi-Shearer K, Razavi H. Global epidemiology and genotype distribution of the hepatitis C virus infection. J Hepatol. 2014; 61(1 Suppl):S45-57.
13. Pybus OG, Markov PV, Wu A, Tatem AJ. Investigating the endemic transmission of the hepatitis C virus. Int J Parasitol. 2007; 37:839–49.
14. Hauri AM, Armstrong GL, Hutin YJ. The global burden of disease attributable to contaminated injections given in health care settings. Int J STD AIDS. 2004;15:7–16.
15. Magiorkinis G, Magiorkinis E, Paraskevis D, et al. The global spread of hepatitis C virus 1a and 1b: a phylodynamic and phylogeographic analysis. PLoS Med. 2009; 6:e1000198.
16. Wyles DL, Luetkemeyer AF. Understanding Hepatitis C Virus Drug Resistance: Clinical Implications for Current and Future Regimens. 2017 (Volume 25, Issue 3).

17. Kwo P, Gitlin N, Nahass R, Bernstein D, Etzkorn K, Rojter S, Schiff E, et al. Simeprevir plus sofosbuvir (12 and 8 weeks) in hepatitis C virus genotype 1-infected patients without cirrhosis: OPTIMIST-1, a phase 3, randomized study. Hepatology. 2016; 64(2):370-80. doi: 10.1002/hep.28467.
18. Lawitz E, Matusow G, DeJesus E, Yoshida EM, Felizarta F, Ghalib R, et al. Simeprevir plus sofosbuvir in patients with chronic hepatitis C virus genotype 1 infection and cirrhosis: A phase 3 study (OPTIMIST-2). Hepatology. 2016;64(2):360-9. doi: 10.1002/hep.28422. Epub 2016 Feb 19.
19. Nelson DR, Cooper JN, Lalezari JP, Lawitz E, Pockros PJ, Gitlin N, et al. ALLY-3 Study Team. All-oral 12-week treatment with daclatasvir plus sofosbuvir in patients with hepatitis C virus genotype 3 infection: ALLY-3 phase III study. Hepatology. 2015;61(4):1127-35. doi: 10.1002/hep.27726. Epub 2015 Mar 10.
20. Sato PA, Maskill WJ, Tamashiro H, Heymann DL. Strategies for laboratory HIV testing: an examination of alternative approaches not requiring Western blot. Bull World Health Organ. 1994;72(1):129-34.
21. Chueca N, Rivadulla I, Lovatti R, Reina G, Blanco A, Fernandez-Caballero JA, et al. Using NS5B Sequencing for Hepatitis C Virus Genotyping Reveals Discordances with Commercial Platforms. PLoS One. 2016;11(4):e0153754. doi: 10.1371/journal.pone.0153754.
22. Cappello JM, Gunasekera A, Gunasekera D, Esfandiari J, Ippolito T. A multicenter performance evaluation of the DPP HIV-1/2 assay for the detection of HIV antibodies in various HIV testing algorithms. J Clin Virol. 2013; 58 Suppl 1:e59-64.

23. R Core Team. (2018) R: A language and environment for statistical computing. R Foundation for Statistical Computing, Vienna, Austria
24. Wickham H. tidyverse: Easily Install and Load the 'Tidyverse'. R package version 1.2.1. https://CRAN.R-project.org/package=tidyverse. 2017.
25. Signorell A. Tools for descriptive statistics. R package version 0.99.25. https://cran.r-project.org/package=DescTools, 2018
26. Warnes GR, Bolker B, Lumley T, Johnson RC. Various R Programming Tools for Model Fitting. R package version 2.18.1. https://CRAN.R-project.org/package=gmodels, 2018.
27. Gower E, Estes C, Blach S, Razavi-Shearer K, Razavi H. Global epidemiology and genotype distribution of the hepatitis C virus infection. J Hepatol. 2014; 61(1 Suppl):S45-57. doi: 10.1016/j.jhep.2014.07.027.
28. Campiotto S1, Pinho JR, Carrilho FJ, Da Silva LC, Souto FJ, Spinelli V, et al. Geographic distribution of hepatitis C virus genotypes in Brazil. Braz J Med Biol Res. 2005;38(1):41-9.
29. Nishiya AS, Almeida-Neto Cd, Romano CM, Alencar CS, Ferreira SC, Di-Lorenzo-Oliveira C, et al. Phylogenetic analysis of the emergence of main hepatitis C virus subtypes in São Paulo, Brazil. Braz J Infect Dis. 2015;19(5):473-8. doi: 10.1016.
30. Alves K, Shafer KP, Caseiro M, Rutherford G, Falcao ME, Sucupira MC, Diaz RS. Risk factors for incident HIV infection among anonymous HIV testing site clients in Santos, Brazil: 1996-1999. J Acquir Immune Defic Syndr. 2003; 32(5):551-9.
31. Braun DL, Hampel B, Martin E, Kouyos R, Kusejko K, Grube C, et al. High Number of Potential Transmitters Revealed in a Population-based Systematic

Hepatitis C Virus RNA Screening Among Human Immunodeficiency Virus-infected Men Who Have Sex With Men. Clin Infect Dis. 2018.

32. Ingiliz P, Wehmeyer M, Christensen S, et al. High incidence of HCV reinfection in MSM in the DAA era. 25th Conference on Retroviruses and Opportunistic Infections (CROI). 2018. Boston. Abstract 612.

33. Rubbia-Brandt L, Quadri R, Abid K, Giostra E, Malé PJ, Mentha G, et al. Hepatocyte steatosis is a cytopathic effect of hepatitis C virus genotype 3. J Hepatol. 2000;33(1):106-15.

34. Nkontchou G, Ziol M, Aout M, Lhabadie M, Baazia Y, Mahmoudi A, et al. HCV genotype 3 is associated with a higher hepatocellular carcinoma incidence in patients with ongoing viral C cirrhosis. J Viral Hepat. 2011;18(10):e516-22. doi: 10.1111/j.1365-2893.2011.01441.x.

35. Kanwal F, Kramer JR, Ilyas J, Duan Z, El-Serag HB. HCV genotype 3 is associated with an increased risk of cirrhosis and hepatocellular cancer in a national sample of U.S. Veterans with HCV. Hepatology. 2014; 60(1):98-105. doi: 10.1002/hep.27095. Epub 2014.

36. Ferenci P, Brunner H, Laferl H, Scherzer TM, Maieron A, Strasser M, et al. A randomized, prospective trial of ribavirin 400 mg/day versus 800 mg/day in combination with peginterferon alfa-2a in hepatitis C virus genotypes 2 and 3. Hepatology. 2008; 47(6):1816-23.

37. Manns M, Zeuzem S, Sood A, Lurie Y, Cornberg M, Klinker H, et al. Reduced dose and duration of peginterferon alfa-2b and weight-based ribavirin in patients with genotype 2 and 3 chronic hepatitis C. J Hepatol. 2011; 55(3):554-563.

38. Foster GR, Hézode C, Bronowicki JP, Carosi G, Weiland O, Verlinden L, et al. Telaprevir alone or with peginterferon and ribavirin reduces HCV RNA in patients with chronic genotype 2 but not genotype 3 infections. Gastroenterology. 2011;141(3):881-889.e1. doi: 10.1053/j.gastro.2011.05.046.

39. Curry MP, O'Leary JG, Bzowej N, Muir AJ, Korenblat KM, Fenkel JM, et al. Sofosbuvir and Velpatasvir for HCV in Patients with Decompensated Cirrhosis. N Engl J Med. 2015;373(27):2618-28.

40. McHutchison JG, Everson GT, Gordon SC, Jacobson IM, Sulkowski M, Kauffman R, McNair L, et al. Telaprevir with peginterferon and ribavirin for chronic HCV genotype 1 infection. N Engl J Med. 2009; 360(18):1827-38. doi: 10.1056/NEJMoa0806104.
